# Folate deficiency induced H2A ubiquitination to lead to downregulated expression of genes involved in neural tube defects

**DOI:** 10.1186/s13072-019-0312-7

**Published:** 2019-11-13

**Authors:** Pei Pei, Xiyue cheng, Juan Yu, Jinying Shen, Xue Li, Jianxin Wu, Shan Wang, Ting Zhang

**Affiliations:** 1 Beijing Municipal Key Laboratory of Child Development and Nutriomics, Capital Institute of Pediatrics, Beijing, 100020 China; 2 Graduate Schools of Peking Union Medical College, Beijing, 100730 China; 3 Department of Biochemistry and Molecular Biology, Shanxi Medical University, Taiyuan, 030001 Shanxi China; 4 School of Engineering Technology, Beijing Normal University, Zhuhai, 519085 Guangdong China; 5 School of Clinical Medical, Weifang Medical University, Weifang, 261053 Shandong China; 6 Institute of Basic Medical Sciences, Chinese Academy of Medical Science, Beijing, 100730 China

**Keywords:** Neural tube defect, Histone ubiquitination, Folate antagonist methotrexate, Mouse double minute 2 homolog (Mdm2, MDM2), Neural tube closure-related genes

## Abstract

**Background:**

Neural tube defects (NTDs) are common congenital malformations resulting in failure of the neural tube closure during early embryonic development. Although it is known that maternal folate deficiency increases the risk of NTDs, the mechanism remains elusive.

**Results:**

Herein, we report that histone H2A monoubiquitination (H2AK119ub1) plays a role in neural tube closure. We found that the folate antagonist methotrexate induced H2AK119ub1 in mouse embryonic stem cells. We demonstrated that an increase in H2AK119ub1 downregulated expression of the neural tube closure-related genes *Cdx2*, *Nes*, *Pax6*, and *Gata4* in mouse embryonic stem cells under folate deficiency conditions. We also determined that the E3 ligase Mdm2 was responsible for the methotrexate-induced increase in H2AK119ub1 and downregulation of neural tube closure-related genes. Surprisingly, we found that Mdm2 is required for MTX-induced H2A ubiquitination and is recruited to the sites of DSB, which is dependent on DNA damage signaling kinase ATM. Furthermore, folic acid supplementation restored H2AK119ub1 binding to neural tube closure-related genes. Downregulation of these genes was also observed in both brain tissue of mouse and human NTD cases, and high levels of H2AK119ub1 were found in the corresponding NTDs samples with their maternal serum folate under low levels. Pearson correlation analysis showed a significant negative correlation between expression of the neural precursor genes and H2AK119ub1.

**Conclusion:**

Our results indicate that folate deficiency contributes to the onset of NTDs by altering H2AK119ub1 and subsequently affecting expression of neural tube closure-related genes. This may be a potential risk factor for NTDs in response to folate deficiency.

## Background

Neural tube defects (NTDs) are severe malformations that originate during early embryonic development and result in failure of the neural tube closure. Both genetic and environmental factors contribute to NTDs [1]. Maternal folate is one of the best-studied dietary factors closely related to neural tube closure (NTC). Worldwide fortification of food with folic acid has resulted in a significant reduction in the prevalence of NTDs [2]. Particular attention has been paid to carbon metabolism, especially the finding of maternal supplementation with folic acid has reduced the risk of NTDs. Environmental factors may influence NTC through a direct effect on the biology of embryonic metabolism. Deficiency of specific folates at the cellular level may be responsible for NTDs as a result of disturbed bioavailability of folates. Folate one-carbon metabolism comprises a complex network of interlinked reactions that mediates transfer of 1-carbon groups to several biosynthetic processes [3] and it focuses particularly on the requirement for methylation reactions in neural tube closure. Abnormal thymidylate and purine biosynthesis have been identified in mouse NTD models and in a proportion of NTD cases [4–6]. Current data suggest that folate deficits likely represent only a moderate fraction of NTD risk, and the mechanisms underlying human NTDs remain elusive [7, 8]. In the prevention and treatment of NTDs with folic acid, anti-folate drugs used during pregnancy had deleterious effects and increased the incidence of NTDs and other congenital defects [9]. As an inhibitor of dihydrofolate reductase, methotrexate (MTX) prevents the conversion of folate to its active form and inhibits nucleotide biosynthesis [10]. Previous studies have shown that MTX inhibits neuronal differentiation in neural stem cells and that folate supplementation attenuates this effect [11].

There is growing awareness that gene–gene and gene–environment interactions are involved in the occurrence of NTDs [12–14]. More than 200 genes are known to cause NTDs in mice, and human NTDs are even more complicated, being multifactorial and polygenic [15, 16]. However, only few of the NTD-associated genes identified in mice are confirmed to be involved in humans [17], and genetic polymorphisms account for only a small proportion of cases. In fact, multiple NTD-causing genes arrange in a particular pathway in NTDs [17]. Recently, potential causative factors for human NTDs have involved deregulation of gene expression through epigenetic mechanisms [18]. Mouse NTD models have indicated that histone modifications might control the expression of patterning molecules that are essential for NTC. Since histone-modifying enzymes consume key metabolites, it is thought that they interpret the metabolic state of a given cell by altering chromatin modification patterns. Polycomb repressive complexes 1 and 2 (PRC1 and PRC2) take part in NTC [19]. The catalysis by PRC1 of H2A monoubiquitination at lysine119 (H2AK119ub1) is a critical component in mediating Polycomb group (PcG) silencing in developmental lineage genes to promote stemness [20, 21]. Histone demethylase UTX is required for NTC in PRC2-mediated H3K27 trimethylation (H3K27me3) [22]. H2AK119ub1 is emerging as a prominent contributor to genetic and molecular etiology in neurodevelopmental disorders [23]. Notably, mouse NTD models have shown that regulation of ubiquitination plays a vital role during NTC [24]. Recent studies have reported that Mdm2 (mouse double minute 2 homolog) is physically associated with EZH2 on chromatin, enhancing H3K27me3 and H2AK119ub1 at its target genes. Removal of Mdm2 stimulates Ring1B/RNF2 and further induces cell dysdifferentiation. Folate-mediated 1-carbon metabolism is related to epigenetic modifications. It affects DNA methylation and is associated with NTDs. This metabolism also controls the fate of neural precursor cell during development and differentiation of embryonic stem cells (ESCs) [25–27]. This is consistent with the highly dynamic yet coordinated cellular and molecular programs that drive changes in morphology, patterning, proliferation, and differentiation during NTC. The observation of cranial NTDs among knock-out acetyltransferase p300 or Gcn5 suggested that activity of histone acetylase is essential for NTC [28, 29].

On the basis of findings that epigenetic regulators play key roles in mouse NTC and may be affected by folic acid [30–32], it is likely that genome-wide analyses will reveal epigenomic changes associated with NTDs. In animal models, advanced technologies allow researchers to evaluate the transcriptional profile of specific cell types and correlate this with changes in DNA methylation, histone marks, and higher order chromatin states. Future studies incorporating information on folic acid status should help to define potential folate-mediated changes, with a particular focus on the genes known to be necessary for NTC. Relation between previous reports showed that DNA hypomethylation of long interspersed nucleotide elements and risk of NTDs. The folate pathway is particularly important because altered patterns of histone modification regulate gene function and stability, in turn leading to congenital malformations. It has been demonstrated that increased H3K27me2 in cultured neural crest cells and neural tube explants from splotch (Pax3) mutant embryos resulted in development of NTDs [33]. This finding showed that folate rescued H3K27me2 and was associated with *Hes1* and *Neurog2* promoters, thereby affecting gene transcription. However, to date only a few studies have addressed the possible role of folate level on the relationship between histone ubiquitination and ESC differentiation in ongoing occurrence of NTDs. This raises the possibility that the relationship between folic acid supplementation or folate 1-carbon metabolism and the risk of NTDs may be mediated in part through their effects on methylation.

Folate deficiency facilitates recruitment of upstream binding factor to hotspots of DNA double-strand breaks (DSBs) [34] of ribosomal (r)RNA genes and promotes their transcription. Furthermore, spontaneous DSBs in cells under folate deficiency conditions were shown to be located exclusively within rRNA gene units, representing an H3K4me1 hallmark. Enrichment of H3K4me1 at the hotspots of DSB regions enhances the recruitment of upstream binding factor to rRNA genes, resulting in an increase in transcription of rRNA genes [35]. Our previous study indicated that folate deficiency attenuated H3K79me2, affecting its regulate activation of target genes, some of which were known to be associated with NTDs, and interrupting early embryo development. Our results suggested that higher levels of homocysteine (Hcy) contribute to the onset of NTDs through upregulation of histone H3K79Hcy, leading to abnormal expression of selected NTC-related genes. Since folate levels contribute to the accumulation of DSBs in the genome, the effects of folate metabolism disorder on the relationship between DSBs in H2AK119ub1 and its transcriptional regulation in NTC genes are unknown.

In this study, we showed that the folate antagonist MTX induced H2AK119ub1 in mouse ESCs. We detected that an increase in H2AK119ub1 level downregulated the expression of NTC-related genes, including *Cdx2*, *Nes*, *Pax6*, and *Gata4* in cells under folate deficient conditions. We also demonstrated that the E3 ligase Mdm2 is responsible for the MTX-induced increase in H2AK119ub1 and downregulated NTC-related genes. Surprisingly, Mdm2 is required for MTX-induced H2A ubiquitination and is recruited to the sites of DSB, which is dependent on DNA damage signaling kinase ATM. Furthermore, folic acid supplementation results in restored H2AK119ub binding to NTC-related genes. In addition, downregulation of NTC-related genes was observed in both brain tissue of mouse and human NTD cases, and low levels of folate found in the corresponding maternal serum samples. Our results provide strong evidence that folate deficiency induces aberrant H2AK119ub1, which is linked to abnormal expression of NTC-related genes and subsequently NTDs. This research further extends our understanding of aberrant epigenetic modification of NTC-related genes in NTDs.

## Results

### Decreased expression of neural precursor marker genes in MTX-treated mouse ESCs

**Fig. 1:**
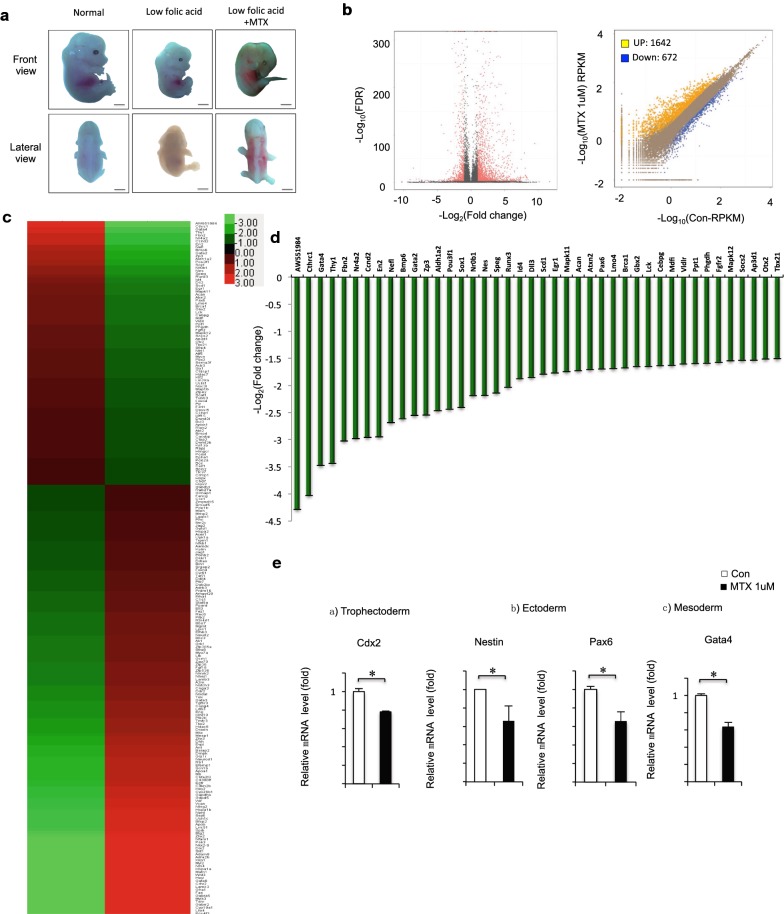
**a** Low folate and MTX induced craniorachischisis in C57BL/6 mouse embryos at 13.5 day. **b** Control vs MTX treatment in mESC. The total RNA was analyzed using RNA-seq. A total of DEGs showing differential expression were identified in the APN-treatment group (*P *< 0.01). **c** Unsupervised hierarchical clustering of MTX-treated cells. **d** DEGs have a statistically significant change with RNA-seq analysis. **e** NTC-related genes Cdx2, Nestin, Pax6 and Gta4 mRNA extracted from mESCs treated with MTX was assessed by RT-qPCR. MTX 1 μM, 12 h. *GAPDH* was used as control. Data are shown as mean (*n* = 3). *P < 0.05, by Student’s *t* test

Folate antagonists (such as MTX) are compounds that antagonize the function of folic acid. When MTX is administered maternally, mouse offspring have NTDs, including spina bifida, exencephaly, and anencephaly [36, 37]. Previous studies have shown that folate deficiency does not lead to NTDs in mice. Therefore, we explored the potential link between a low-folate diet with MTX injection, and the failure of NTC in in vivo experiments using a mouse model. As shown in Fig. 1a (left), the normal mouse appears smooth and round, with a clear zygomatic arch, slender tail, and completely closed neural tube. Morphological changes in the normal mouse embryo were prominent at embryonic day (E) 13.5 (equivalent to days 22–23 to days 26–30 of gestation in humans). Then, a low folate-induced mouse model was established: normal C57 mice received the low-folate diet for at least 2 weeks. Those pregnant mice were simply on low folate diet cannot deliver properly. Offspring of mice on the low-folate diet had a small and hypoplastic brain vesicle, accompanied by overall growth retardation and varying degrees of abnormality in tectology (Fig. 1a, middle). Next, a mouse with weight gain of 0.8–1 g was injected intraperitoneally with MTX at 1.5 mg/kg of body weight on E7.5. This dose of MTX has previously been shown to cause NTDs in our lab; therefore, we used it to construct a folate deficiency-induced NTD mouse model. In the mice injected with 1.5 mg/kg MTX, the morphology of mouse embryos differed significantly compared with that of control embryos; the defects observed were mainly spina bifida. The mouse embryo survival rate was 19.2% (10/52), the resorption rate was 9.6% (5/52), the growth retardation rate was 35% (18/52), and the malformation rate was 36.5% (19/52). After MTX treatment under low-folate conditions, 36.5% of embryos had NTDs (Fig. 1a, right).

To identify the alterations in gene expression profile accompanied by MTX-induced changes, we performed RNA-seq on mESCs treated with 1 μM MTX. By comparing libraries from control and MTX-induced mESC samples, we identified a significant number of differentially expressed genes, including 1642 upregulated and 672 downregulated genes (Fig. 1b; Additional file 1: Table S1). Further analysis with Gene Ontology (GO) and Kyoto Encyclopedia of Genes and Genomes (KEGG) pathways indicated that these differentially expressed genes were enriched in GO terms related to multiple biological processes, molecular functions, and signaling pathways of MTX-treated cells (Additional file 2: Figure S1A), and KEGG pathways (Additional file 2: Figure S1B and Additional file 3: Table S2). Unsupervised clustering analysis revealed numerous differentially regulated genes under MTX treatment, with enrichment of gene sets involved in development and morphogenesis (Fig. 1c and Additional file 2: Figure S1C). Overall, 829 differentially expressed genes had > twofold changes (723 upregulated and 126 downregulated) following MTX treatment (Additional file 4: Table S3). Of the 42 genes strongly downregulated under MTX treatment, especially NTC-related genes associated with neural precursor stage (Fig. 1d). To determine the role of MTX in the neural plate stage in ESC, we determined the effect of MTX treatment on mESC using real-time quantitative PCR (RT-qPCR). The RT-qPCR analysis revealed that several NTC-related genes were downregulated in MTX-treated mESC, including neuronal filament genes *Cdx2* and *Nes* and genes encoding the pro-neuronal transcription factors *Pax6* and *Gata4*, consistent with RNA-seq data (Fig. 1e). Taken together, our data suggest that the MTX treatment of mESC resulted in changes in gene expression pattern and decreased expression of neural precursor marker genes.

### MTX-induced H2AK119ub1 binding to NTC-related genes in mESCs

**Fig. 2:**
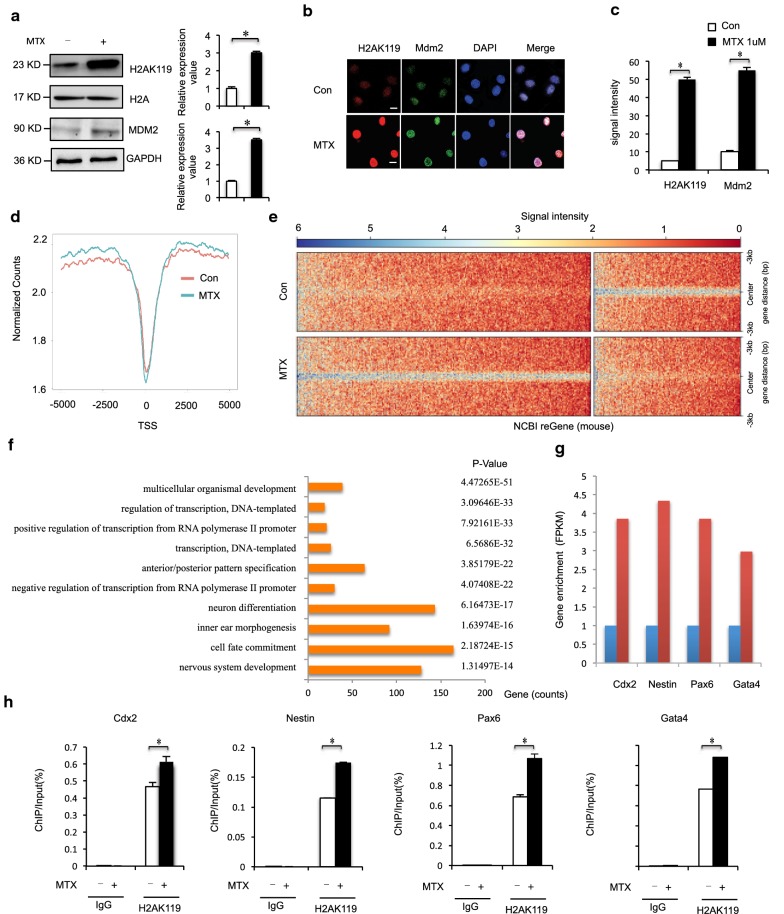
**a** Mouse ESCs were harvested under MTX (1 μM) treatment for 12 h, and analyzed by western blot. Numbers at the bottom were generated by quantification (ImageJ) of the H2AK119ub1 signal normalized to the H3 signal and MDM2 signal normalized to the GAPDH signal. **b**, **c** Immunostaining using MDM2 antibodies formed discrete foci in mESC cells for 12 h after exposure to MTX (1 μM) and colocalized with H2AK119ub1. The analysis was confined to the transfected cells. The error bars represent the SEM. **d**, **e** H2AK119ub1 ChIP-Seq, aggregated around TSSs. H2AK119ub1 ChIP-Seq in mESC cells 12 h after exposure to MTX (1 μM). **f** Functional network of enriched genes with H2AK119ub1 peaks. DAVID method was used to do functional annotation clustering for biological process annotations of genes with H2AK119ub1 peaks. **g** ChIP-seq (upper) analysis on Cdx2, Nestin, Pax6 and Gata4 genes in control and MTX-treated mESC. **h** ChIP assays of H2AK119ub1 were performed using mouse ESCs treated with 1 μM MTX for 12 h. Mouse IgG was used as control. Enrichment of NTC-related genes Cdx2, Nestin, Pax6 and Gata4 promoters was measured by qPCR. Data are shown as mean (*n* = 3). *P < 0.05, by Student’s *t* test

We next evaluated whether cellular levels of MTX could influence levels of H2AK119ub1. Mouse ESCs (mESCs) were treated with a half-maximal inhibitory concentration of MTX after 12, 24, and 48 h [40]. Western blot analysis of extracted histones revealed that H2AK119ub1 levels increased with increasing concentration of MTX dosage at 1 μM within 12 h (Fig. 2a). Our data suggest that elevated H2AK119ub1 may underlie the failure of NTC during early development because of a functional disturbance of folate-mediated 1-carbon metabolism. Since Mdm2 enhances H2AK119ub1, we examined whether Mdm2 enhanced MTX-induced H2AK119ub1. Interestingly, H2AK119u1 levels increased concomitantly accompanied by increased RING finger E3 ligase Mdm2 protein levels in mESC (Fig. 2a). We also found that H2AK119ub1 and Mdm2 were significantly colocalized to nuclear foci after exposure to MTX (Fig. 2b, c). To further explore the importance of histone H2AK119ub1 during neural system development, ChIP-seq was carried out in MTX-treated mESCs. By analyzing equal numbers of reads from H2AK119ub1, a total of 1295 peaks from 3255 genes were detected using an anti-H2AK119ub1 antibody, scanning through the entire mouse genome. ChIP-seq of H2AK119ub1 target genes showed that the enrichment level of H2AK119ub1 was significantly increased in MTX-treated mESCs compared with controls (Fig. 2d, Additional file 5: Table S4). ChIP-seq analysis identified a remarkable overall change of H2AK119ub1 near transcription start sites in MTX-treated cells (Fig. 2e). ChIP GO analysis showed that among genes targeted by H2AK119ub1 there was a bias toward genes related to the nervous system. Of the top 10 GO groups with enriched peaks, the top 5 were related to anterior/posterior pattern specification, the top 7 were related to neuron differentiation, and the top 10 were related to the nervous system (Fig. 2f, Additional file 6: Tables S5 and Additional file 7: S6). The group of genes with the most-enriched peak groups was those involved in nervous system development, followed by those involved in the generation of neurons and neurogenesis. ChIP-seq analysis showed that the H2AK119ub1 enrichment levels of the four NTC-related genes were higher in MTX-treated mESC than in untreated mESC (Fig. 2g). Next, we investigated the extent of H2AK119ub1 by ChIP at transcriptional activation of NTC-related genes in MTX-treated mESCs. As shown in Fig. 2h, binding of these NTC-related genes (*Cdx2*, *Nes*, *Pax6*, and *Gata4*) was sequentially enhanced after MTX treatment. In contrast, no changes were observed in binding of H2AK119ub1 to IgG loci (Fig. 2h). Thus, MTX treatment led to upregulation of H2AK119ub1 and subsequently to enhanced binding of H2AK119ub1 to promoters of *Cdx2*, *Nes*, *Pax6*, and *Gata4*. Collectively, our data indicate that during MTX treatment, the enrichment of H2AK119ub1 on NTC-related genes (*Cdx2*, *Nes*, *Pax6*, and *Gata4*) was increased, their expression level decreased, and the overall level of histone H2AK119ub1 was increased. These data indicated that increased binding of H2AK119ub1 to NTC-related genes was induced by MTX, resulting in repression of NTC-related genes.

### Mdm2 is required for regulation of H2AK119ub1 on NTC-related genes under MTX treatment

**Fig. 3:**
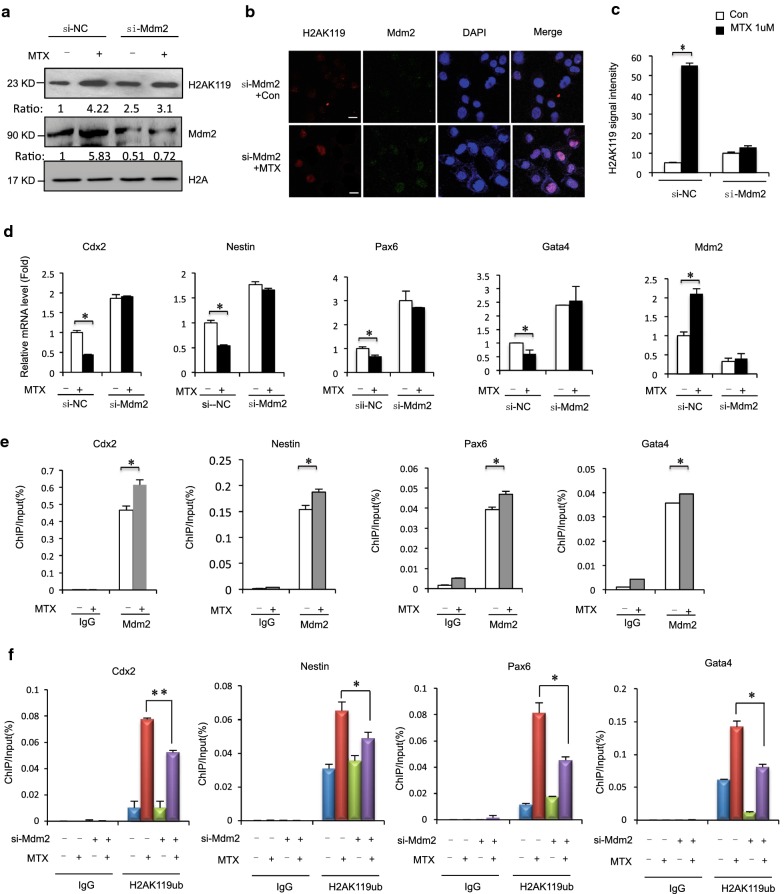
**a** Mouse ESCs were transfected with siRNA-MDM2 or control, and 24 h later treated with MTX 12 h and harvested for Western blot analysis. **b**, **c** Mouse ESCs were transfected with siRNA-MDM2, and 24 h later treated with MTX for 12 h. Immunostaining using MDM2 antibodies formed discrete foci and colocalized with H2AK119ub1. Analysis was confined to transfected cells. Error bars represent the SEM. **d** Mouse ESCs were transfected with siRNA-MDM2 or control, and 24 h later treated with MTX for 12 h. Expressions of the indicated genes were quantified by RT-qPCR. All values were normalized to GAPDH at mRNA level in the same sample. Data are shown as mean (*n* = 3). *P < 0.05, by Student’s *t* test. **e** ChIP assays of MDM2 were performed using mouse ESCs treated with 1 μM MTX for 12 h. Mouse IgG was used as control. Enrichment of NTC-related genes Cdx2, Nestin, Pax6 and Gata4 gene promoters was measured by RT-qPCR. Data are shown as mean (*n* = 3). *P < 0.05, by Student’s *t* test. **f** Mouse ESCs were transfected with siRNA-MDM2 or control, and 24 h later treated with MTX for 12 h. Cells subjected to ChIP analysis with antibodies specific for H2AK19ub1. Immunoprecipitated DNA was quantified by RT-qPCR with primers specific for the 5′ transcribed regions of the indicated genes. Data are shown as mean (*n* = 3). *P < 0.05, by Student’s *t* test

Previous genetic analyses demonstrated that PcG target gene loci are often regulated by PRC1 and PRC2 complex, and that histone-modifying activity (H2AK119u1 and H3K27me3, respectively) is critical to gene repression. The RING finger E3 ligase Mdm2 is physically associated with EZH2 and enhances H2AK119ub1 and H3K27me3 at its target genes [41]. Next, we studied the effects of Mdm2 depletion on H2AK119ub1 following MTX treatment. Mouse embryonic carcinoma (F9) cells were transfected with short interfering (si)RNA-Mdm2 and control siRNA, with and without MTX treatment, and levels of H2AK119ub1 were monitored. MTX induction increased H2AK119ub1, and Mdm2 depletion decreased MTX-induced H2AK119ub1 (Fig. 3a). We also found that Mdm2 depletion attenuated MTX-induced H2AK119ub1 foci (Fig. 3b, c). Remarkably, MTX significantly induced downregulation of the NTC-related genes *Cdx2*, *Nes*, *Pax6*, and *Gata4*. Downregulation of these genes was attenuated after Mdm2 knockdown in F9 cells (Fig. 3d). Next, we examined the binding of Mdm2 to promoters of the selected transcriptional activation in *Cdx2*, *Nes*, *Pax6*, and *Gata4* using a ChIP-qPCR assay. As shown in Fig. 3e, although no changes were detected in Mdm2 binding to promoter regions of IgG, increased Mdm2 binding to the promoter regions of the transcriptional activation of NTC-related genes was evident in MTX-treated mESCs. Thus, H2AK119ub1 compromised with transcription associated with NTC-related genes after downregulation of Mdm2 in MTX-treated F9 cells. Furthermore, partial depletion of Mdm2 decreased both basal and MTX-induced levels of H2AK119ub1 within those genes (Fig. 3f). Overall, our findings indicated that downregulation of Mdm2 interfered with MTX-induced H2AK119ub1 on NTC-related genes in F9 cells, suggesting that Mdm2 is required for expression of *Cdx2*, *Nes*, *Pax6*, and *Gata4* during MTX treatment.

### Mdm2 to DNA damage sites and contributes to MTX-induced H2AK119ub1

**Fig. 4:**
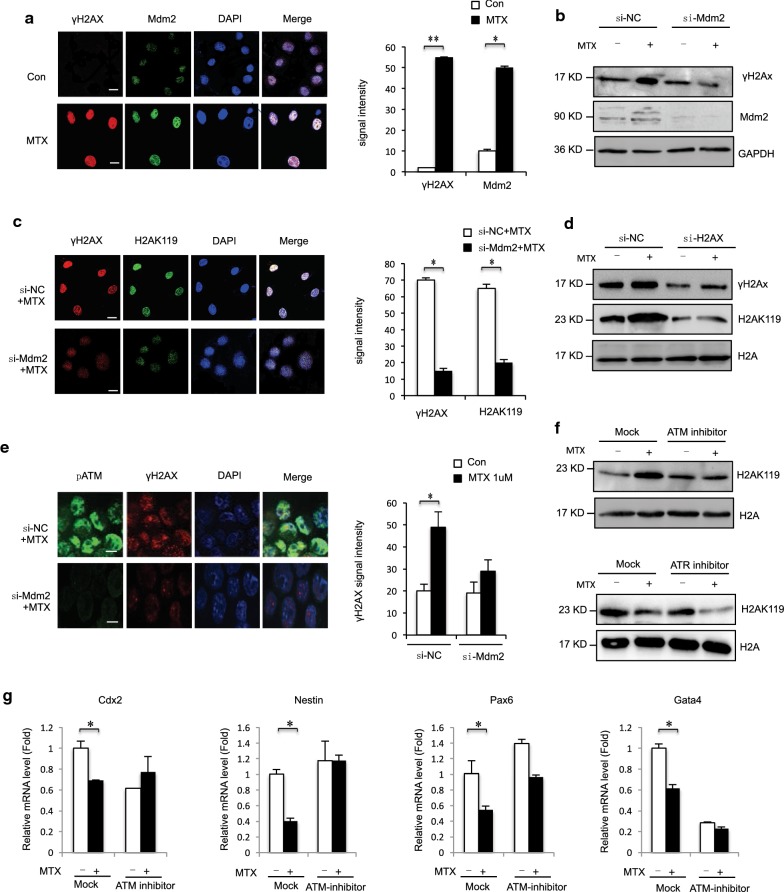
**a** Immunostaining using MDM2 antibodies formed discrete foci in mESC cells 12 h after exposure to MTX (1 μM) and colocalized with γH2AX. The analysis was confined to the transfected cells. The error bars represent the SEM. **b** F9 cells exposure to MTX (1 μM) 12 h. γH2AX were detected in MDM2-depleted F9 cells at levels comparable to those observed in control siRNA-treated cells and analyzed by western blot. **c** Immunostaining using H2AK119ub1 antibodies formed discrete foci in mESC cells 12 h after exposure to MTX (1 μM) and colocalized with γH2AX. The analysis was confined to the transfected cells. Error bars represent the SEM. **d** F9 cells exposure to MTX (1 μM) 12 h. H2AK119ub1 were detected in γH2AX -depleted F9 cells at levels comparable to those observed in control siRNA-treated cells and analyzed by western blot. **e** Normal foci for phosphorylated ATM were formed 12 h after exposure in MTX (1 μM) F9 cells in which MDM2 was downregulated. The analysis was confined to the transfected cells. Error bars represent the SEM. **f** F9 cells treatment with MTX (1 μM) 12 h and/or ATM inhibitor KU55933 (3 mM) for 12 h/ATR inhibitor 24 h and analyzed by western blot indicated antibody. **g** F9 cells treatment with MTX (1 μM) 12 h and/or ATM inhibitor KU55933 (3 mM) for 12 h/and expression levels of NTC-related genes Cdx2, Nestin, Pax6 and Gata4 analyzed by RT-qPCR

Previously, we showed that MTX induced significant changes in DNA DSBs in mESC [35]. H2A ubiquitination plays various roles in cellular processes such as transcriptional regulation and DNA damage repair [42–44]. One of the initial targets of DSB signaling is the phosphorylation of histone H2A variant H2AX. It accumulates in chromatin surrounding sites of DSBs to generate structures called H2AX foci [45]. To investigate the role of Mdm2 and H2AK119ub1 in DSB repair, we immunostained particular protein using indicated antibody. We found that Mdm2 was localized to nuclear foci after exposure to MTX, and that Mdm2 foci significantly colocalized with phosphorylated H2AX (γH2AX) foci (Fig. 4a). We next investigated γH2AX by downregulation of Mdm2 under MTX treatment. Results showed that Mdm2 depletion attenuated the MTX-induced phosphorylation of H2AX (Fig. 4b). Immunostaining showed that MTX-induced phosphorylation of H2AX was attenuated after Mdm2 knockdown (Fig. 4c). Similarly, H2AK119 was affected in cells where H2AX was downregulated (Fig. 4d). Phosphorylation of H2AX by ATM (ataxia telangiectasia mutated), ATR (ATM- and Rad3-related), and DNA-dependent protein kinase (DNA-PK) occurs in response to DSBs and represents histone modifications induced by DNA damage [46, 47]. Phosphorylated ATM recruited by γH2AX formed normal MTX-induced foci and colocalized with γH2AX in Mdm2-depleted cells (Fig. 4e). Furthermore, to determine the role of ATM and ATR activation in Mdm2 recruitment, experiments were performed with specific ATM and ATR inhibitors (ATMi: KU55933, ATRi: VE-821) [48]. Treatment with the ATMi led to loss of signal for phosphorylated ATM-serine 1981 and a significant reduction in H2AX phosphorylation at the sites of induced DNA damage. H2AK119ub1 was decreased by treatment with KU55933, a kinase inhibitor of ATM, and was unaffected by treatment with ATRi (Fig. 4f). Furthermore, Mdm2 was phosphorylated at the SQ/TQ motif after exposure to MTX (data not shown). Treatment with ATMi (KU55933) attenuated the MTX-induced significant downregulation of the NTC-related genes *Cdx2*, *Nes*, *Pax6*, and *Gata4* in F9 cells (Fig. 4g). Therefore, MTX-induced H2AK119ub1 by Mdm2 associated with ATM.

### Supplementation with folinic acid attenuates H2AK119ub1 induced by MTX and affects H2AK119ub binding to NTC-related genes

**Fig. 5:**
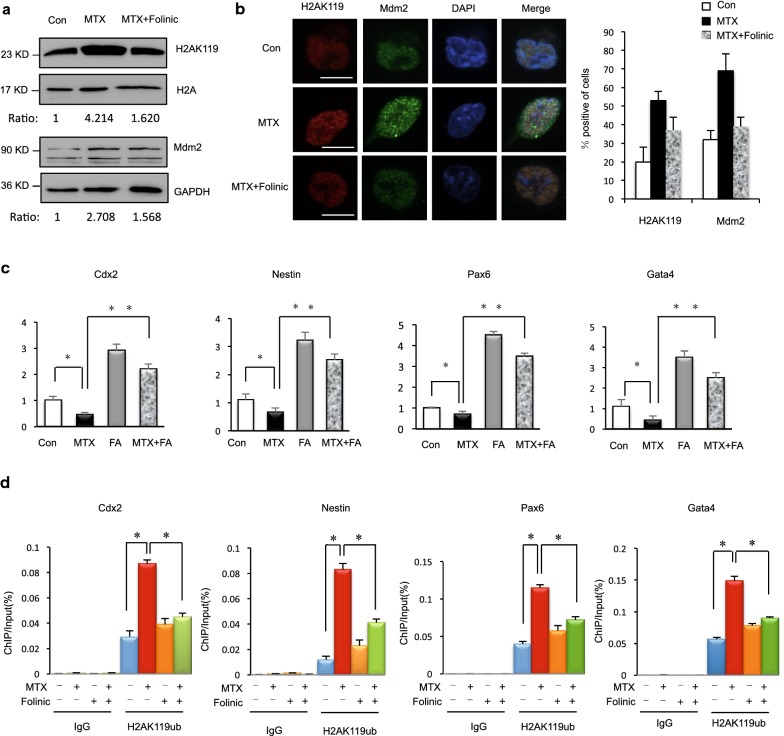
**a** mESCs were cultured in complete medium, complete medium with 1 μM MTX, and supplementary folinic acid (50 mg/L) for 24 h and analyzed by western blot indicated antibody. **b** Immunostaining assay in mESCs by 1 μM MTX treatment and after folinic acid supplementation. MDM2 antibodies formed discrete foci in mESC cells and colocalized with H2AK119ub1. Analysis was confined to the transfected cells. Error bars represent the SEM. **c** Expression levels of NTC-related genes Cdx2, Nestin, Pax6 and Gata4 in mESCs cultured in complete medium with either 1 μM MTX for 24 h and folinic acid supplementation or both conditions determined by RT-qPCR. Data are shown as mean (*n* = 3). *P < 0.05, **P < 0.01, by Student’s *t* test. **d** H2AK119ub1 states at representative in NTC-related genes Cdx2, Nestin, Pax6 and Gata4 after folinic acid supplementation compared with MTX treatment alone, as determined by ChIP-qPCR. Data are shown as mean (*n* = 3). *P < 0.05, by Student’s *t* test

With regard to non-genetic factors, failure of NTC can be induced by a wide variety of teratogenic agents in rodent models [33], whereas a smaller number of non-genetic factors are definitively associated with NTDs in humans. Folinic acid supplementation can increase utilization of folate. In fact, rapidly dividing cells in neural tube development require a large number of nucleotides to be synthesized to facilitate DNA replication. At the cellular level, folinic acid supplementation affects cell differentiation, proliferation, and junction formation in the neurosphere. Our previous research indicated that supplementation with folinic acid affects DSBs and mainly targets the G_1_ phase in mESC. Folinic acid supplementation could be used as an antidote to decrease MTX-induced toxicity. However, less is known about whether folinic acid antagonizes MTX-induced H2AK119ub1 and mESC differentiation. In this study, we supplemented folinic acid (50 mg/L) to mESCs in culture medium pretreated with MTX. The H2AK119ub1 level was increased by MTX but attenuated by folinic acid supplementation (Fig. 5a). Next, we found that the foci of Mdm2 and H2AK119ub1 were significantly reduced in cells treated with folinic acid (Fig. 5b). These results further indicate that the induction of H2AK119ub1 by MTX in mESCs is intimately associated with folate deficiency. Next, we performed comparable experiments that either put MTX and folic acid or both conditions. First, the results showed that the NTC-related genes *Cdx2*, *Nes*, *Pax6*, and *Gata4* were downregulated under MTX treatment (Fig. 5c Lane 1 vs. Lane 2) and this effect was reversed by folinic acid supplementation (Fig. 5c Lane 2 vs. Lane 4). Interestingly, folic acid also increased the NTC-related genes *Cdx2*, *Nes*, *Pax6*, and *Gata4* (Fig. 5c Lane 1 vs. Lane 3). After folic acid treatment, the expression of NTC-related genes is bigger than in both MTX and folinic acid (Fig. 5c Lane 3 vs. Lane 4). Second, we examined the binding of H2AK119ub1 to promoters of the same genes using ChIP-qPCR in two groups. ChIP analysis showed that MTX treatment led to an increase in H2AK119ub1 within transcribed regions of *Cdx2*, *Nes*, *Pax6*, and *Gata4* (Fig. 5d Lane 5 vs. Lane 6), whereas H2AK119ub1 within those genes was markedly reduced after folinic acid supplementation (Fig. 5d Lane 6 vs. Lane 8). The results also showed that H2AK119ub1 within those genes was markedly reduced only folinic acid supplementation (Fig. 5d Lane 6 vs. Lane 7). In contrast, we observed insignificant changes in the binding of H2AK119 to IgG loci (Fig. 5d Lane 1–Lane 4). Decreased H2AK119ub1 occupancy in *Cdx2*, *Nes*, *Pax6*, and *Gata4* after folinic acid supplementation indicated an association of H2AK119ub1 with these neural precursor marker genes involved in differentiation in mESCs under low folate status. This result implied that folinic acid supplementation reduced H2AK119ub1 induced by MTX and affected H2AK119ub1 binding to NTC-related genes.

### MTX caused induced levels of H2AK119ub1 in the NTD mice

**Fig. 6:**
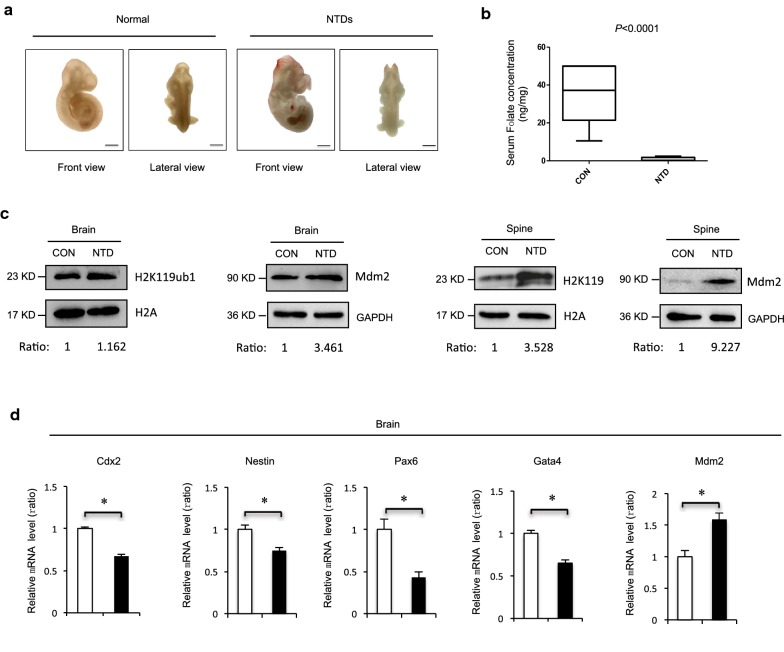
**a** MTX induced Myelomeningocele in C57BL/6 mouse embryos at 13.5 day. **b** Folate content in brain serum of normal and MTX-induced mouse NTDs was harvested at E13.5, and analyzed by Elisa. Data are mean ± S.D. (*n* = 3). *P < 0.05, by Student’s *t* test. **c** Cranial neural tissue of normal and MTX-induced mouse NTDs was harvested at *E* 13.5, and analyzed by western blot. Aliquots of total lysates were immunoblotted to indicate antibody. **d** Neural precursor marker genes cdx2, nestin, pax6, gata4 and mdm2 extracted mRNA in cranial neural tissue of MTX-induced mouse NTDs was measured by RT-qPCR. Data are mean ± S.D (*n* = 3). *P < 0.05, by Student’s *t* test

Folates may be a potential risk to NTDs because of their roles in nucleotide synthesis. MTX is a folate antagonist that competitively inhibits dihydrofolate reductase to interfere with purine and pyrimidine biosynthesis, thus affecting DNA replication and cell proliferation [38, 39]. We constructed a mouse model via intraperitoneal injection of MTX (1.5 mg/kg) at embryonic day 7.5 with low diet folate. As shown in Fig. 6a, the appearance of normal mice is smooth and rounded, with clear zygomatic arch, slender tail and completely closed neural tube. The failure of closure at the level of the hindbrain/cervical boundary at this stage leads to craniorachischisis. Next, we determined whether the maternal serum level of folate decreased in our folate-deficient mouse model. Folate levels were significantly decreased in the maternal serum under MTX induction (Fig. 6b), suggesting that MTX resulted in folate deficiency during early pregnancy, affecting neurogenesis in fetuses. Western blot was performed to compare H2AK119ub1 levels in samples from the control and MTX groups. Higher levels of histone H2AK119ub1 were detected in samples from the MTX-treated group with phenotypes of NTDs. H2AK119ub1 was markedly increased in MTX-induced cranial neural tissue of spine from E13.5, indicating that abnormal H2AK119ub1 expression may lead to the occurrence of NTDs in mice (Fig. 6c). Embryonic developmental decisions happen in three stages: first, there is the orchestrated movement of cells to either the embryonic or extraembryonic line and then to the mesendoderm or ectoderm line, and finally the pluripotent cells become ectodermal or neural precursors before termination. Morphogenesis is the process by which the three-dimensional shape is formed and the three germ layers build a complex structure. Next, we detected NTC-related genes in mouse embryo NTD samples. The RT-qPCR indicated that mRNA levels of *Cdx2*, *Nes*, *Pax6*, and *Gata4* were significantly reduced in mouse NTD embryos compared with controls (Fig. 6d; *p* < 0.05). Together, these results indicated that H2AK119ub1 levels increased concomitantly with the reduction of neural precursor lineage specification, which was accompanied by increased Mdm2 protein levels in MTX-induced mouse NTDs.

### Aberrant H2AK119ub1 with decreased expression in low-folate NTD fetuses

**Fig. 7:**
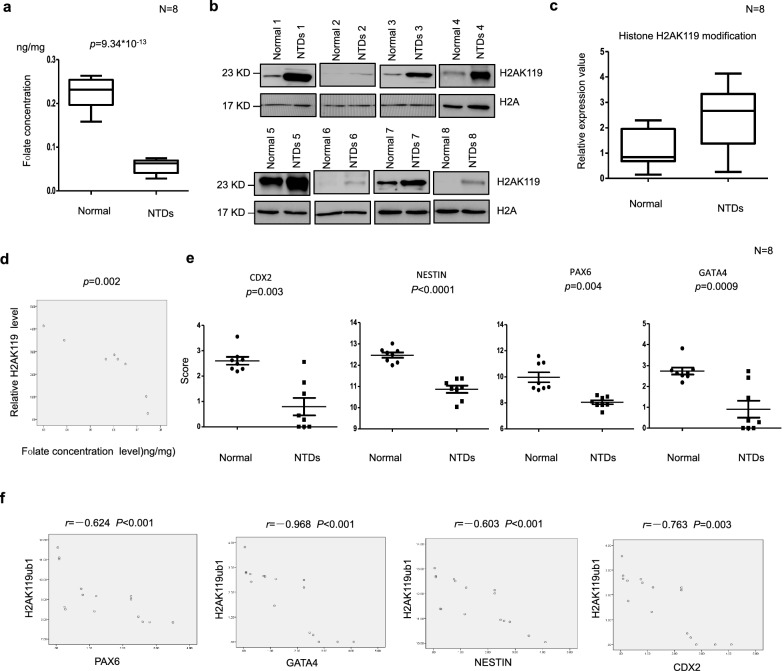
**a** Elisa assay detected folate content in brain tissue of normal fetus and fetus with NTDs. **b** Detection of H2AK119ub1 in brain tissues from normal fetus and low-folate NTDs with low folate maternal serum. H2A was used as a loading control. Data are mean ± SD. (*n* = 3),*P < 0.05, by Student’s *t* test. **c** Quantification of the western blotting H2AK119ub1 signal intensity shown in scatter plot. Column normals: *n* = 8; Column NTDs: *n* = 8; *p < 0.05, by Student’s *t* test. NTDs vs. normal brains. **d** Pearson *R*- and *P* value for normalized H2AK119ub1 levels versus low folate content in low-folate NTDs fetuses (*n* = 8). Data are presented as mean ± SEM. ***P = 0.002. **e** The mRNA expression of NTC-related genes CDX2, NESTIN, PAX6 and GATA4 in the NTDs fetuses brain, determined by Nanostring. Data are mean ± SD (*n* = 10),*P < 0.05, by Student’s *t* test. **f** Pearson’s correlation analysis between H2AK119ub1 expression and NTC-related genes CDX2, NESTIN, PAX6 and GATA4

Neural tube closure requires cooperation of several mechanisms, such as neural crest cell migration, proliferation, and differentiation. During neurogenesis, insufficient neuronal differentiation causes NTDs. We evaluated brain tissue of human fetuses with NTDs and folate levels in the corresponding maternal serum samples. Twelve pairs including fetuses with spina bifida were selected, and the age and sex of fetuses were closely matched, as summarized in Table 1. Maternal serum folate levels were lower in pairs with fetuses affected by spina bifida than in controls (Fig. 7a). Next, we detected H2AK19ub1 in the fetuses with NTDs. Their maternal serum was detected under low folate. Western blot analysis of eight fetuses with spina bifida compared with their gestational age- and sex-matched controls revealed that H2AK119ub1 was increased in most samples from fetuses with spina bifida (Fig. 7b). The average level of H2AK119ub1 expression (normalized to H2A) was found to be significantly higher in NTD samples (*p* < 0.05; Fig. 7c). Importantly, levels of H2AK119ub1 correlated well with maternal folate levels (Fig. 7d). These results indicate upregulation of H2AK119ub1 levels in fetuses with NTDs. We also examined NTC-related genes in low-folate NTD fetuses. Results showed that expression of *CDX2*, *NES*, *PAX6*, and *GATA4* was significantly decreased in human NTD samples (Fig. 7e; *p* < 0.05). Correlation analysis showed significant negative correlations between H2AK119ub1 and *CDX2*, *NES*, *PAX6*, and *GATA4* (Fig. 7f). These results indicated that H2AK119ub1 was altered with decreased expression of NTC-related genes in low-folate NTD fetuses. Our data indicate that low folate levels may result in an increase in the level of H2AK119ub1, which may suppress the transcription of *CDX2*, *NES*, *PAX6*, and *GATA4*, leading to NTDs.

**Table 1:** Additional information of the human normal and NTD samples

Sample type	Tissue	Folate level (ng/mg)	Gender	Gestational weeks
Normal	Brain	0.222133333	Female	17
Normal	Brain	0.194133333	Female	18
Normal	Brain	0.2264	Male	20
Normal	Brain	0.257866667	Female	18
Normal	Brain	0.2362	Male	19
Normal	Brain	0.15918396	Male	16
Normal	Brain	0.24133	Male	18
Normal	Brain	0.204933333	Male	16
NTD	Brain	0.064133333	Female	25
NTD	Brain	0.0746	Female	25
NTD	Brain	0.030333333	Female	32
NTD	Brain	0.065133333	Female	34
NTD	Brain	0.061866667	Male	31
NTD	Brain	0.056733333	Male	21
NTD	Brain	0.068933333	Female	25
NTD	Brain	0.043933333	Male	39

## Discussion

A protective effect of folic acid on the occurrence and recurrence of NTDs has been clearly demonstrated. Abnormal 1-carbon metabolism has been implicated in the occurrence of NTDs. Maternal folate deficiency during pregnancy is associated with an increased risk of NTDs. However, the mechanisms underlying the effect of folic acid on NTDs remain to be elucidated. It seems that folate deficiency alone is insufficient to cause NTDs but can give rise to offspring with NTDs when combined with multiple genetic and environmental factors. We showed that increased H2AK119ub1 on neural precursor genes in mESCs under folate deficiency interfered with cell differentiation. Supplementation with folinic acid rescued potential proliferation in neural precursor marker genes in MTX-treated mESCs. It is possible that H2AK119ub1 in folate-mediated rescue affects transcription of neural precursor marker genes during the early developmental stage. Moreover, we found that decreased expression of NTC-related genes was accompanied by aberrant H2AK119 expression in human fetuses NTDs under folate deficiency.

Neurogenesis is regulated by genetic and epigenetic mechanisms. Genetic, non-genetic, epigenetic, and environmental factors all contribute to the prevalence of NTDs. Environmental factors may influence NTC through a direct effect on metabolism. Much evidence has shown that folate 1-carbon metabolism nutrients may affect epigenetic regulation, thereby influencing susceptibility to NTDs through altered gene expression. A causal link between histone methylation and nutritional status has also been demonstrated, where folate and methionine deficiency are associated with reduced histone methylation and can lead to changes in gene expression. In the present study, we explored pathways from the 1-carbon metabolism intermediate Hcy to the onset of NTDs based on several key observations, including the demonstrated increase in Hcy levels in women who give birth to infants with NTDs, the histone modifications KHcy and H3K79Hcy, and the well-established altered expression of NTC genes in NTDs. Our previous studies found that folate deficiency affected DNA breakage through histone H3K4me1, leading to regulation of transcription of rRNA genes during the early stage of development and offering new insights into the etiology of NTDs. In this study, we showed that folate deficiency affected H2AK119ub1. Our data showed that H2AK119ub1 was increased in the cranial neural tube of embryos with MTX-induced NTDs compared with wild-type controls, suggest that upregulation of H2AK119ub1 was involved in open NTDs (Figs. 6c, 7b). We also found that increased H2AK119ub1 altered expression of NTC genes in folate-deficient NTD samples (Fig. 7). The PcG proteins form large complexes that repress gene expression through H3K27me3 and H2AK119ub1. These histone modifications are essential in postnatal development and stem cell maintenance in the mouse [49]. We focused on H2AK119ub1 because PcG proteins mediate suppression of PcG target genes, mammalian body patterning, posterior development, and possibly identity maintenance in embryonic stem cells. Folate deficiency leads to delayed embryonic development and growth retardation in early embryonic progression [44]. Recent studies have shown that an epigenetic component of folate-mediated rescue could affect cell proliferation, including transcription of *Hes1* and other genes involved in early development, and thereby rescue posterior neural tube development. Indeed, epigenetic mechanisms likely govern all aspects of the central nervous system, including neuronal gene expression [43], especially in cell differentiation during neuro-embryogenesis.

To select potential epigenetically altered genes, we focused on the neural precursor marker genes *Cdx2*, *Nes*, *Pax6*, and *Gata4*. Recent reports have shown that H2AK119ub1 alters the activities of these genes. *Cdx2* and *Nes* are responsible for neuronal filament growth, *Pax6* is critical in pro-neuronal transcription, and *Gata4* is involved in maintenance of stem cell characteristics and sensory neurogenesis. In our study, inhibition of folate metabolism was specifically targeted to inactivate these selected NTC-related genes (*CDX2*, *NES*, *PAX6*, and *GATA4*) in low-folate fetuses (Fig. 7d). The upregulation by H2AK119ub1 of these NTC-related genes could be a molecular regulatory event involved in NTC. This study suggested that H2AK119ub1 repressed NTC-related genes in MTX-treated mESC (Fig. 2h). Folinic acid supplementation may attenuate H2AK119ub1 by decreasing levels of Mdm2 (Fig. 5a). Subsequently decreased Mdm2-repressed chromatin marks on the NTC-related genes at its promoter regions, resulting in increased expression of NTC-related genes.

Reports indicate that inadequate proliferation is associated with occurrence of NTDs [50, 51]. In mice, over 200 mutant genes are involved in open NTDs, but much less are known about genetic causation of human NTDs. The nested expression patterns of *Pax6* demarcate the forebrain at the neural plate stage. Abnormal expression of *Pax6* results in severe NTDs, in which the neural tube remains open from the midbrain to lower spine. The intermediate filament protein nestin is an important marker of multipotent neural stem cells. When H2AK119ub1 functioned on NTC-related genes *PAX6* and *NES*, it progressed neural tube closure in an amphibian embryo. Folate, especially of maternal folic acid supplementation, reduces the risk of non-folic-acid-resistant NTDs. In our study, we demonstrated folate metabolism inhibition on transcription neural precursor-specific genes during early embryonic development in mESC. The occurrence of isolated NTDs at the cranial or caudal level in humans and different mouse models suggests the possible involvement of region-specific mechanisms, depending on expression of different genes; all stages have ubiquitous requirements. In mice, NTDs can be modeled by exposure to certain reagents. Our results suggest that the neural precursor-specific genes are involved in different phenotypes of NTDs. Thus, in different tissues, NTD phenotypes may be associated with prevention of neuronal differentiation. Together, these data indicated that H2AK119ub regulated NTC-related genes at its promoter regions under folate deficiency may result in NTDs.

Mdm2 forms an active heterodimer that catalyzes H2AK119ub1 and H3K27me3 by the physical association of Mdm2 and PRC2. Mdm2 regulates PRC2 target genes by promoting positive feedback between H3K27me3 and H2AK119ub1, which explains why H2AK119ub1 increases with increased Mdm2 expression with MTX treatment [52]. Our previous research identified a role of MTX in changes in DNA DSBs in mESC. Furthermore, spontaneous DSBs in mammalian cells under folate deficiency have been located to H3K4me1 [35]. H2AK119ub1 is induced locally at the sites of damaged DNA. We reported here on a new function of H2AK119ub1 in DNA damage response. Under MTX treatment, H2AK119ub1 colocalized with ATM and γH2AX. Mdm2 undergoes specific modifications in response to replicative stress of DNA damage and is associated with damage response factors such as the Mre11-Rad50-Nbs1 (MRN) complex [46, 47]. We demonstrated MTX-induced increased expression of Mdm2 in mESCs (Fig. 2a) and found that Mdm2 was bound to NTC-related genes under folate deficiency conditions (Fig. 3e). Downregulation of H2AK119ub1 attenuated expression of NTC-related gene when Mdm2 was depleted (Fig. 3f). Folate plays an important role in neuroplasticity and maintenance of neuronal integrity.

Our analyses provide novel insight into neural precursor marker genes and potential regulation of H2A modification under abnormal folate metabolism. We identified an association of H2AK119ub1 with NTC-related genes during early embryonic development, suggesting a dynamic interplay of Mdm2 in fine-tuning stem cell proliferation and differentiation. Dysregulation of H2AK119 in NTDs was involved in downregulation of NTC-related genes under folate deficiency. A better understanding of H2AK119ub1 may improve identification and diagnosis of NTDs. These results showed that abnormal histone ubiquitination regulates neural precursor marker genes in NTDs, and it provides evidence of regulation of histone ubiquitination followed by folinic acid supplementation. Our study may indicate one mechanism by which folate may affect human neural tube disorders.

Neural tube defects are common congenital malformations resulting from the failure of NTC during early embryonic development. Maternal folate level is one of the best-studied dietary factors relative to NTC. We showed that H2A monoubiquitination (H2AK119ub1) plays a role in NTC. We found that the folate antagonist MTX induced H2AK119ub1 in a mouse NTD model and in mESC. Using ChIP-seq and RNA-seq assays, we demonstrated that an increase in H2AK119ub1 level downregulated the expression of the NTC-related genes *Cdx2*, *Nes*, *Pax6*, and *Gata4* in mESCs under conditions of folate deficiency. We also showed that the E3 ligase Mdm2 was responsible for the MTX-induced increase in H2AK119ub1 and downregulated NTC-related genes. Furthermore, folic acid supplementation restored H2AK119ub binding to NTC-related genes. Downregulation of NTC-related genes was observed in brain tissues of fetuses with NTDs, and the maternal serum were all under low folate condition, along with aberrant H2AK119ub1 levels. This evidence suggests that folate deficiency contributes to the onset of NTDs by altering H2AK119ub1 and subsequently affecting expression of NTC-related genes. This may be a potential risk factor for NTDs in response to folate deficiency.

## Conclusions

In this study, we showed that the folate antagonist MTX induced H2AK119ub1 in mouse ESCs. We detected that an increase in H2AK119ub1 level downregulated the expression of NTC-related genes, including *Cdx2*, *Nes*, *Pax6*, and *Gata4* in cells under folate deficiency conditions. We also demonstrated that the E3 ligase Mdm2 is responsible for the MTX-induced increase in H2AK119ub1 and downregulated NTC-related genes. Surprisingly, Mdm2 is required for MTX-induced H2A ubiquitination and is recruited to the sites of DSB, which is dependent on DNA damage signaling kinase ATM. Furthermore, folic acid supplementation results in restored H2AK119ub binding to NTC-related genes. In addition, downregulation of NTC-related genes was observed in both brain tissue of mouse and human NTD cases, and low levels of folate and higher levels of H2AK119ub1 were found in the corresponding maternal serum samples. Our results provide strong evidence that folate deficiency induces aberrant H2AK119ub1, which is linked to abnormal expression of NTC-related genes and subsequently NTDs. This research further extends our understanding of aberrant epigenetic modification of NTC-related genes in NTDs.

## Methods

### Animals

C57BL/6 mice (6–8 weeks, 18–20 g), were provided by Jinmyung animal laboratory, and feed under SPF (Specific Pathogen Free) growing condition, approved facility on a 12-h light/dark cycle. Male and female C57BL/6 mice were fed on low folate diet for more than 4 weeks. Sexually matured individuals mated overnight; vaginal plug was detected at 8:00 am in the following morning, which designated as E0.5 days. NTDs mouse models were induced by intraperitoneal injection with 1.5 mg/kg (body weight) of MTX (Sigma, USA) on E7.5. On E13.5, pregnant mice were euthanized and killed by cervical dislocation. Embryos were dissected and placed in ice-cold tubes, DEPC-treated PBS. All procedures involving animal handling were in compliance with institutional guidelines on the care of experimental animals.

### Cell culture and differentiation assays

Mouse embryonic stem cells (mESCs) Sv/129 were maintained in Dulbecco’s modified Eagle’s medium (DMEM, Gibco, USA) supplemented with 0.1 mM β-mercaptoethanol (Invitrogen, Carlsbad, USA), 0.1 mM non-essential amino acids (Invitrogen, Carlsbad, USA), 0.1 mM glutamate (Invitrogen, Carlsbad, USA), 15% fetal bovine serum (Gibco, USA), and 1000 U/ml mouse leukemia inhibitory factor (Millipore, Billerica, USA), cultured in culture dishes coated with 0.2% gelatin (Invitrogen, Carlsbad, USA). Cells were placed in humidified incubator with 37 °C, 5% CO_2_ passaged every 2–3 days. F9 cells were cultured in Dulbecco’s modified Eagle medium with 10% FBS. mESCs were suspended to form embryoid body (EB), with 20% serum. mESCs were treated with a concentration gradient of MTX for 12, 24, 48 h. Cells were incubated at 37 °C/5% CO_2_ and passaged every 2 days.

### RNA interference

SiRNA Mdm2: 1-GGAACAAGAGACUCUGGUUTT; 2-AACCAGAGUCUCUUGUUCCTT.

### Western blotting

The blots were incubated with the primary antibody, mouse anti- H2AK119ub monoclonal antibody (1:1500, CST, USA) and mouse anti-H2A monoclonal antibody (1:1,500,000, CST, USA) overnight at 4 °C, and then incubated with secondary anti-rabbit HRP-conjugated antibody (1:5000, CST, USA) for 1 h at room temperature. The blots were developed with SuperSignal West Pico Chemiluminescence Substrate (Thermo, USA) and quantitated on densitometer (Bio-Rad, Universal HoodII, USA) using Quantity One software.

### RNA-Seq and data analysis

Total RNA was extracted from cells with Trizol (Invitrogen) according to the manufacturer’s protocol and purified by mRNA enrichment. mRNA poly(A) tails then enriched with oligodT magnetic bead. rRNAs were removed. rRNA hybridized with DNA probe. RNaseH chose to digest DNA/RNA hybridization chain. DNase-digested DNA probe resulted in purified RNA. The cDNA libraries were constructed for each pooled sample with VAHTSTM Total RNA-seq (H/M/R) according to the manufacturer’s instructions. Final cDNA libraries were created by PCR purification and enrichment, then quantified by Agilent2200. The tagged cDNA libraries were pooled in equal ratio and used for 150 bp paired-end sequencing in a single lane of Illumina HiSeq Xten by NovelBio Corp. Laboratory. NovelBrain Cloud Analysis platform was used to analyse high-throughput sequencing data. All RNA-Seq reads were mapped to mouse genome-using TopHat2. Transcript abundance was quantified using Cufflinks and annotations from Ensembl release 70, and FPKM (fragments/kb of transcript/million fragments mapped) values were calculated. To minimize dispersion effect by low-FPKM values, all the FPKM values were modified by addition of 0.1 in log2 transformation. Gene ontology analysis for biological process of the selected genes was performed using Partek Genomic Suite (Ryoka systems).

### ChIP-Seq and data analysis

Before CHIP-sequencing, samples were qualified by Agilent2200. Sample fragments and concentration have to be 100-500 bp and greater or equal to 1 ng/μl. 10^7^ mESC cells were used as ChIP samples. ChIP was done with Simple ChIP Enzymatic chromatin IP kit (9003 s) from Cell Signaling Technology and chromatin was sheared to an average DNA fragment size of 100–300 bp. ChIP-Seq libraries were prepared according to Illumina protocols. Sequencing was done with Illumina NovaSeq 6000. DNA libraries were prepared by NEB Ultra DNA library prep kit under Illumina guide. All ChIP-Seq reads were mapped to the mouse genome (mm9) using Bowtie2 with default parameters. Genomic profiles were generated using IGVtools and were viewed in Integrative Genomics Viewer (IGV). Peaks of H2AK119ub1 ChIP-Seq signals on genome were determined using MACS2 with false-discovery rate as 0.05. Each associated gene for the peaks was determined using Entrez gene annotation with in-house computer program, in which Ash1 l-target genes were defined as genes containing H2AK119ub1-peaks around transcription start site (TSS) within + /24 kb. Datasets for reads/kb/million mapped (RPKM) values of H2AK119ub1 in coding regions of each gene were normalized to 75th percentile.

### Chromatin immunoprecipitation (ChIP) analysis

ChIP assays were performed using the SimpleChIP Enzymatic chromatinIP system (Cell Signaling, California, USA) following the manufacturer’s protocols. Chromatin was prepared, sonicated to DNA segments between 100 and 500 bp and then immunoprecipitated with Ubiquityl-Histone H2A (Lys119) (D27C4) XP^®^ Rabbit mAb (CST, USA). The immunoprecipitated DNA was analyzed by RT-qPCR, which were performed using QuantStudio 7 Flex with SYBR Green detection. The primers used for ChIP assays were shown in Additional file 8: Table S7. Normal Rabbit IgG is used as negative controls in immunoprecipitations. The following equation was used to calculate percent input = 2% × 2^ (CT) 2% input sample − (CT) IP sample).

### RT-qPCR

Total RNA was extracted using the Trizol reagent (Invitrogen, USA), first-strand synthesis was done with RevertAid First Strand cDNA Synthesis Kit (ABM, Canada). Maxima SYBR Green/ROX qPCR Master Mix (ABM, Canada) was used for RT-qPCR and the procedure was as follows: enzyme activation was done at (95 °C, 10 min) × 1 cycle; denature at (95 °C, 35 s) × 35 cycles; annealing at (60 °C, 60 s) x 35 cycles; melting curve at (95 °C, 15 s; 60 °C, 60 s; 95 °C, 15 s; 60 °C 15 s) × 1 cycles; primers were shown in Additional file 8: Table S7.

### Immunofluorescence

For detection of subcellular localization by immunofluorescence, cells were fixed in methanol under − 20° and permeabilized in 0.2% Triton X-100 (PBS) for 20 min, cells were incubated with the indicated Mdm2, H2AK119ub, γH2AX, and pATM antibodies (dilution 1:50; CST, Santa Cruze) for 16 h at 4 °C, followed by incubation with Alexa Fluor-conjugated secondary antibody (dilution 1:200; CST, USA) for 1 h at 25 °C. The nuclei were stained with DAPI (Sigma), and images were visualized with a Zeiss LSM 510 Meta inverted confocal microscope. Colocalization values were calculated from FV10-ASW 3.0 Viewer of Mdm2, H2AK119ub, gH2AX, and ATM.

### Nanostring

The NanoString nCounter was used to detect the number of transcripts in human brain tissues. Total RNA was extracted following the manufacturer’s instructions (miRNeasy Mini Kit, Qiagen) and gene specific probes were designed by the manufacturer (NanoString Technologies). Hybridizations were carried out according to the nCounter Element 24-plex Assay Manual. Approximately 100 ng of each RNA sample was mixed with 20 μl of nCounter Reporter probes in hybridization buffer and 5 μl of nCounter Capture probes for a total reaction volume of 30 μl. The hybridizations were incubated at 65 °C for approximately 16 h, then eluted and immobilized in the cartridge for data collection, which was performed on the nCounter Digital Analyzer. Gene expression data were filtered using quality control (QC) criteria according to the manufacturer’s recommendations. Raw counts of QC-passed samples were normalized using three reference genes as internal controls (GAPDH, CLTC and GUSB). All QC and normalization procedures were performed using nSolver Analysis Software v2.0; all data were log_2_-transformed before further analysis. The Student’s *t* test was used to compare normalized expression values between normal and NTDs.

### Statistical analysis

All the experiments were repeated independently at least twice, and data are presented as mean ± SD. Statistical significance was determined using the Student’s *t* test. A *P* value of < 0.05 was considered to be statistically significant and is presented as *P < 0.05 or **P < 0.01.

### Ethics statement

All animals were handled in strict accordance with the “Guide for the Care and Use of Laboratory Animals” and the “Principles for the Utilization and Care of Vertebrate Animals”, and all animal work was approved by Institutional Animal Care and Use Committee (IACUC) at the Beijing Institute of Radiation Medicine. The study using clinical samples including 8 paired human NTDs and matched normal tissues were approved by department of Lvliang area of Shanxi Province in northern China. Informed consent was obtained from all subjects or their relatives. Human samples were collected and analyzed in accordance with Capital Institute of Pediatrics approval. The Ethics Board of Capital Institute of Pediatrics approved the study protocol. All animal experiments were conducted in compliance with the guidelines of the Institute for Laboratory Animal Research, Capital Institute of Pediatrics.

## Supplementary information

**Additional file 1: Table S1.** RNA-seq assay of all gene changes.

**Additional file 2: Figure S1. A, B** Go and KEGG analysis of the DEGs in indicated group’s protein in MTX treatment mESC. **C** Control vs MTX treatment in mESC (Enlarged image Fig. 1b).

**Additional file 3: Table S2.** RNA-seq assay of KEGG.

**Additional file 4: Table S3.** RNA- seq assay of different gene greater than 2.

**Additional file 5: Table S4.** ChIP-seq assay of peaks.loc.

**Additional file 6: Table S5.** ChIP-seq assay of GO.

**Additional file 7: Table S6.** ChIP-seq assay of KEGG.

**Additional file 8: Table S7.** ChIP-qPCR and qPCR primers.

## Data Availability

Not applicable.

## Abbreviations

ATM: ataxia telangiectasia mutated
ATR: ATM- and Rad3-related
ChIP: chromatin immunoprecipitation
DEGs: differentially expressed genes
DNA-PK: DNA-dependent protein kinase
DSBs: DNA double-strand breaks
DHFR: dihydrofolate reductase
F9: mouse embryonic carcinoma
GO: gene ontology
KEGG: Kyoto Encyclopedia of Genes and Genomes
MTX: methotrexate
H2AK119ub1: H2A monoubiquitylation at lysine119
H3K27me3: H3K27 trimethylation
IC50: half inhibition concentration
mESC: mouse embryo stem cells
NTDs: neural tube defects
NTC: neural tube closure
NT: neural tube
PRCs: polycomb protein complexes
RNA-seq: RNA sequencing
UBF: upstream binding factor
