# Supplementary figures and images for: Folate deficiency induced H2A ubiquitination to lead to downregulated expression of genes involved in neural tube defects
Source: Epigenetics Chromatin. 2019 Nov 13;12:69. doi: 10.1186/s13072-019-0312-7 (PMC6852770; doi:10.1186/s13072-019-0312-7)

## Slide 1
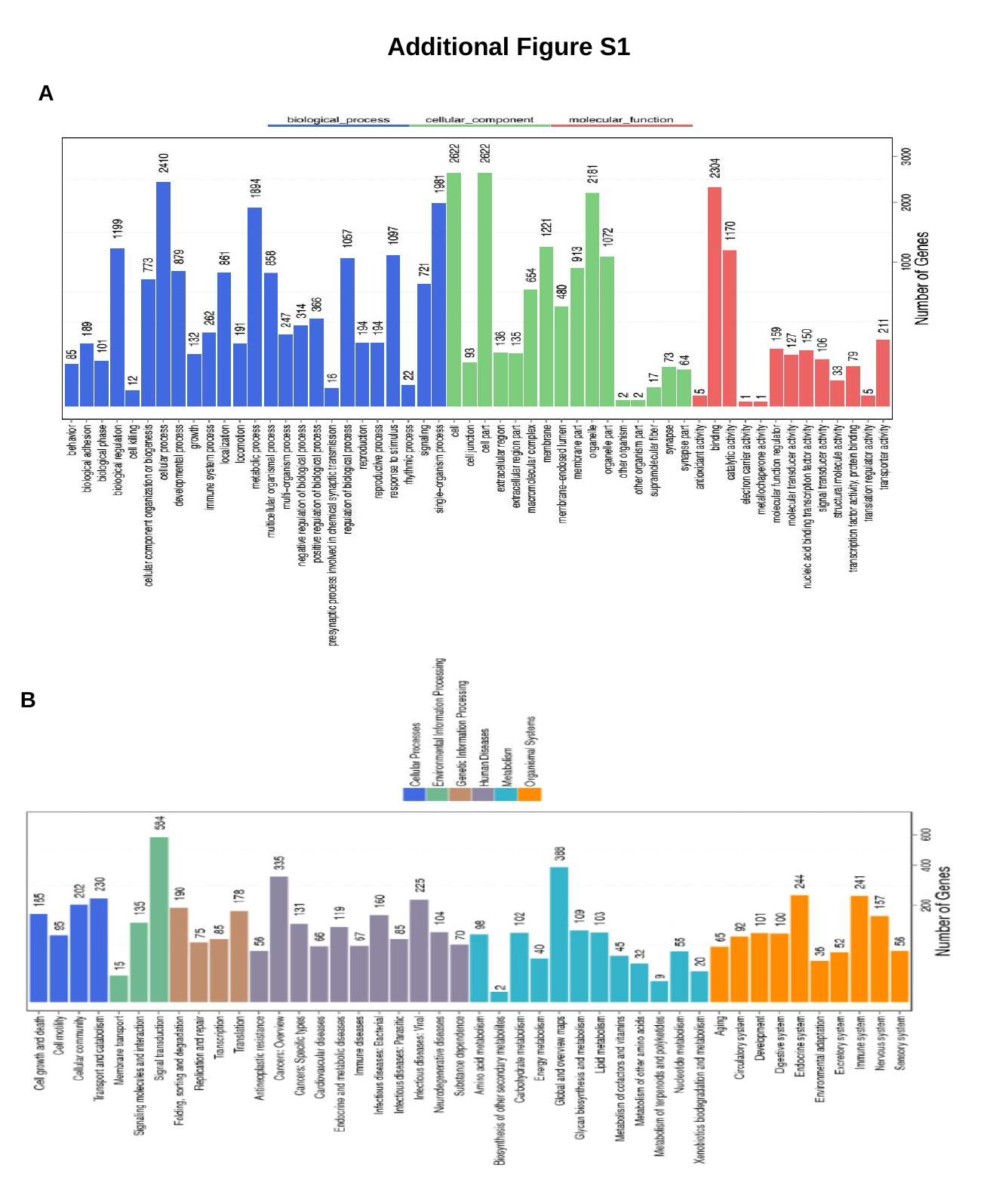

Additional Figure S1
A
B

## Slide 2
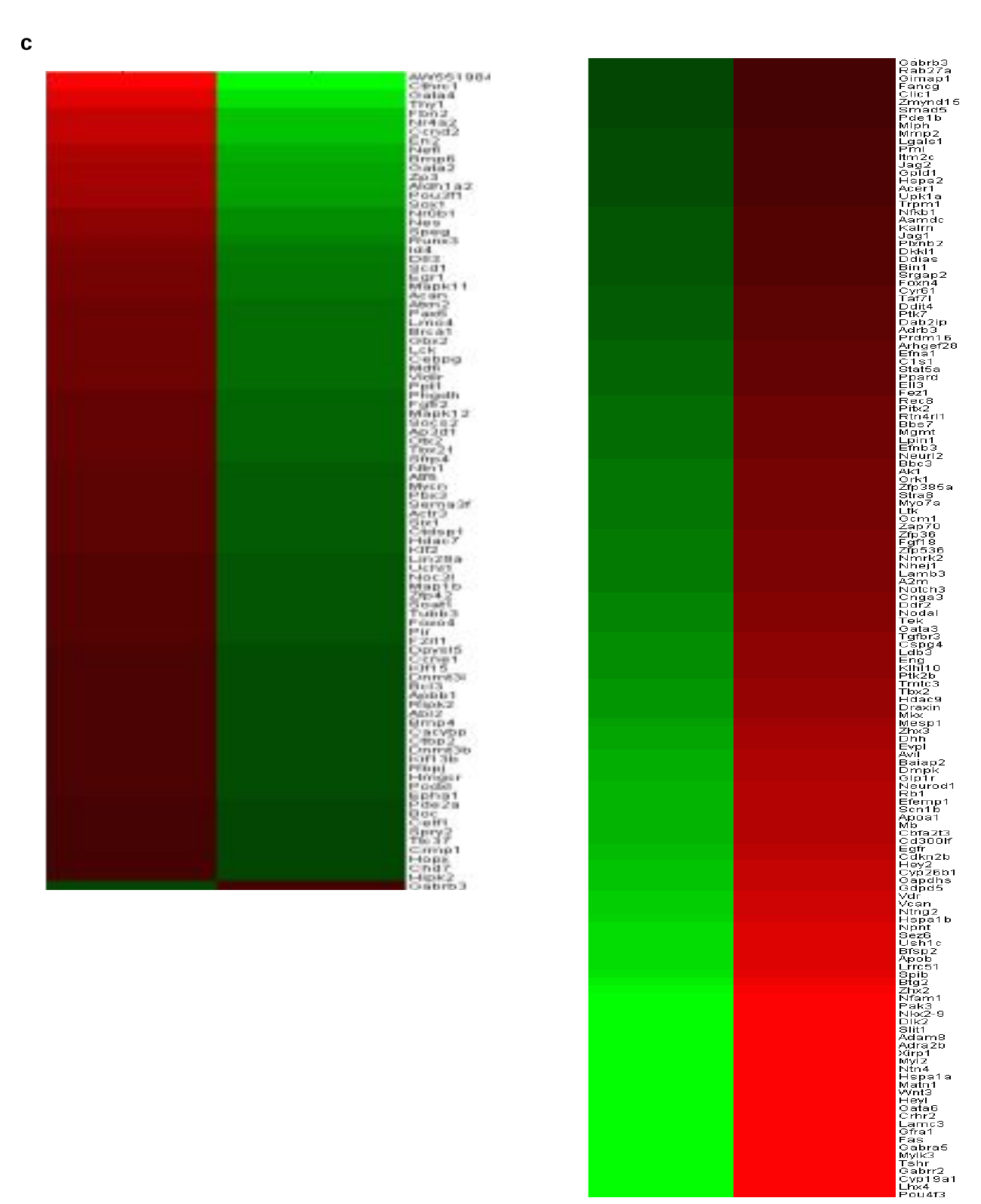

c

Supplement: Supplementary file 2 — Additional file 2: Figure S1. A, B Go and KEGG analysis of the DEGs in indicated group’s protein in MTX treatment mESC. C Control vs MTX treatment in mESC (Enlarged image Fig. 1b). [file 13072_2019_312_MOESM2_ESM.pptx]
